# Analgesic efficacy of ultrasound-guided transversus abdominis plane block in dogs undergoing ovariectomy

**DOI:** 10.3389/fvets.2022.1031345

**Published:** 2022-10-31

**Authors:** Jéssica Sperandio Cavaco, Pablo Ezequiel Otero, Aline Magalhães Ambrósio, Ieda Cristina Boni Neves, Felipe Montanheiro Perencin, Marco Aurélio Amador Pereira, Julia Maria Matera, Denise Tabacchi Fantoni

**Affiliations:** ^1^Department of Surgery, Faculty of Veterinary Medicine and Animal Science, University of São Paulo, São Paulo, Brazil; ^2^Department of Anesthesiology and Pain Management, Facultad de Ciencias Veterinarias, Universidad de Buenos Aires, Buenos Aires, Argentina

**Keywords:** TAP block, pain, analgesia, local anesthesia, bupivacaine, ultrasound, ovariectomy

## Abstract

**Background:**

In medicine, the transversus abdominis plane (TAP) block has been shown as an effective method of analgesia in several surgical procedures. In this context, this prospective, randomized, blinded study aimed to evaluate the analgesic efficacy of TAP block, guided by ultrasound in female dogs submitted to ovariectomy.

**Methods:**

Therefore, 32 animals randomly assigned in two groups (*n* = 16) were used. Groups consisted of TAP block control (TBC) which received water injection (0.2 ml kg^−1^ point), and TAP block bupivacaine (TBB) which received bupivacaine (0.2 ml kg^−1^ point at 0.25%); both groups were submitted to four-point approach. Animals were pre-medicated with acepromazine (0.03 mg kg^−1^) and meperidine (2 mg kg^−1^) IM, propofol was used as anesthetic induction (3–5 mg kg^−1^) IV, and isoflurane was used to maintain. To standardize groups, the animals received a continuous infusion of remifentanil (0.2μg kg^−1^ min) and rocuronium (0.6 mg kg^−1^) IV in the intraoperative period. Variables measured were the heart and respiratory rates, blood pressure, temperature, peripheral oxyhemoglobin saturation, exhaled carbon dioxide concentration, exhaled isoflurane concentration, serum cortisol, analgesia, and sedation. Before the pre-anesthetic medication (Baseline) and 1, 2, 4, 6, and 8 h after extubation, pain and sedation were assessed using a numeric rating scale (NRS), Glasgow composite measure pain scale (GCMPS-SF), and sedation scale. Moreover, serum cortisol was measured at different moments.

**Results:**

The results show that in the intraoperative period, there was no significant difference between groups. After surgery, in TBC, 13 out of 16 animals required analgesic rescue, whereas, in TBB, this occurred only in one animal. Regarding the measurement of serum cortisol, the TBC group showed a significant difference when compared to the baseline time in the traction of the first ovary (*p* < 0.0001), 2 h (*p* = 0.0441), and 8 h (*p* = 0.0384) after extubation. In TBB, cortisol showed a significant increase only in the traction of the first ovary and 2 h after extubation (*p* < 0.0001).

**Conclusion:**

The technique using ultrasound-guided TAP block in two points approach by hemiabdomen with 0.2 ml kg^−1^ bupivacaine 0.25% was effective in providing post-operative analgesia in dogs undergoing ovariectomy.

## Introduction

The transversus abdominis plane (TAP) block aims to promote analgesia of the abdominal wall and parietal peritoneum and has been used as part of a multimodal analgesia protocol for various procedures in humans and animals (1–5).

The TAP block can be realized at two points between the muscle transversus abdominis and the muscle obliquus internus abdominis or three points, being two points between the transversus abdominis and the obliquus internus abdominal and one point between the transversus abdominis and rectus abdominis muscles (6, 7). It consists of depositing local anesthetic in the interfacial plane, and the block should be performed bilaterally to promote analgesia in the midline region (8).

In veterinary medicine, TAP block was described for the first time in 2010 in a Canadian lynx (*Lynx canadensis*) submitted to an exploratory laparotomy to remove a foreign gastric body (9). Subsequently, some studies and reports were developed on dog cadavers (10–14) and in clinical scenarios such as abdominal wall reconstruction (15), mastectomy (3, 16), ovariectomy in cats (5), ovariohysterectomy in dogs (17) and mild and severe abdominal pain secondary to pancreatitis or abdominal surgery (4). However, there is still no consensus in the medical and veterinary medical literature regarding the concentration, volume, and local anesthetic needed to perform this technique (14, 18). Nonetheless, among the studies conducted with animals, bupivacaine is the most used drug (3, 4, 9, 15, 16).

It is noteworthy that this technique can present complications such as intraperitoneal injection, puncture of abdominal organs, incomplete block (6), liver laceration (19), allergic reaction to the local anesthetic, bowel hematoma, intraperitoneal injection associated with risk of injury to abdominal viscera and toxicity by local anesthetics, mainly if bilateral injections are performed (20).

Pain scales are validated tools to assess the effectiveness of different post-operative analgesic treatments. Another valuable instrument that offers good evidence concerning the sympathetic stimulation promoted by nociception is the adrenocortical responses and the increases in plasma cortisol level, a biomarker already used in animal pain studies (21, 22).

To our knowledge, there is one study in which the TAP block was assessed for its effectiveness in providing adequate post-operative analgesia in dogs undergoing ovariohysterectomy. It showed sufficient analgesia (17). Thus, this study aims to evaluate the effectiveness of this technique in providing post-operative analgesia in dogs submitted to ovariectomy. Two pain scales and plasma cortisol levels were utilized to assess the animals. We hypothesized that TAP block would reduce the need for rescue analgesia in the post-operative period.

## Materials and methods

The Institutional Ethics Committee approved the study on the Use of Animals (Protocol: 7979270717) and developed it at its Veterinary Teaching Hospital. Written informed consent was obtained from the animals' owners before the start of the procedures.

### Animals

Thirty-two healthy female dogs, aged between 6 months and 3 years, scheduled to undergo elective ovariectomy were selected. The inclusion criteria for the study were an ASA (The American Society of Anesthesiologists) physical status I and a body condition score between 4 and 5 on a scale of 1–9 (23). The exclusion criteria were hematological, cardiological, and neurological abnormalities, those with systemic and chronic diseases, and patients with any sign of infection of the abdominal wall and skin and anomalies in the puncture site. In addition, female dogs with pseudocyesis, hostile behavior, pregnant, in estrus, with signs of the pain of any intensity and origin, and those who received any previous medication, were excluded from the trial.

### Surgical and anesthetic procedure

All patients underwent clinical evaluation 1 week before the surgical procedure, including complete physical examination, vaginal cytology, blood cell count, and serum biochemical analyzes.

Animals were fasted for at least 8 h for food and 4 h for water. The pre-anesthetic evaluation consisted of measuring heart rate (HR), respiratory rate (*f*_R_), non-invasive blood pressure (NIBP), capillary refill time, degree of hydration, mucous membrane color, and rectal temperature (RT).

Dogs were pre-medicated with acepromazine (0.03 mg kg^−1^) and meperidine (2 mg kg^−1^) by the intramuscular route (IM). After 20 min, aseptic venous access was instituted by a catheter fixed in the cephalic vein, and lactated Ringer's solution started at 5 ml kg^−1^ h. For induction of anesthesia, propofol (3–5 mg kg^−1^) intravenously (IV) was administered until adequate muscle relaxation and loss of interdigital and laryngotracheal reflex was achieved.

Intubation was accomplished with an endotracheal tube of the appropriate size. Anesthetic maintenance was performed with an end-tidal isoflurane concentration of 1.0–1.2% and a continuous rate infusion (CRI) of remifentanil (0.2μg kg^−1^ min), and a bolus of rocuronium (0.6 mg kg^−1^) to guarantee adequate analgesia and muscle relaxation, respectively. A rebreathing circular circuit and controlled mechanical ventilation by the pressure mode, with a peak inspiratory pressure of 8–10 mmHg to achieve an adequate tidal volume (Fabius Plus^®^, Drager do Brasil, Barueri, SP). The respiratory rate was adjusted to maintain end-tidal carbon dioxide between 35–45 mmHg, and the inspired oxygen (air-oxygen mixture) fraction was 60%. These variables were continuously monitored with a sidestream gas analyzer and capnography. At the end of the surgery, animals received neostigmine (0.044 mg kg^−1^) and atropine (0.044 mg kg^−1^) intravenously.

### Experimental groups

Animals were randomly distributed in two groups of 16 (http://www.randomization.com), as follows: TBC (TAP block control group), which received water for injection (0.2 ml kg^−1^) per injection site and administered in the four points approach as described below. TBB (TAP block bupivacaine group) received bupivacaine (0.2 ml kg^−1^) at 0.25% per injection point, also performed at the four points. The dilution of the bupivacaine was performed with water injection immediately before injection.

### Ultrasound-guided TAP injection

**Figure 1:**
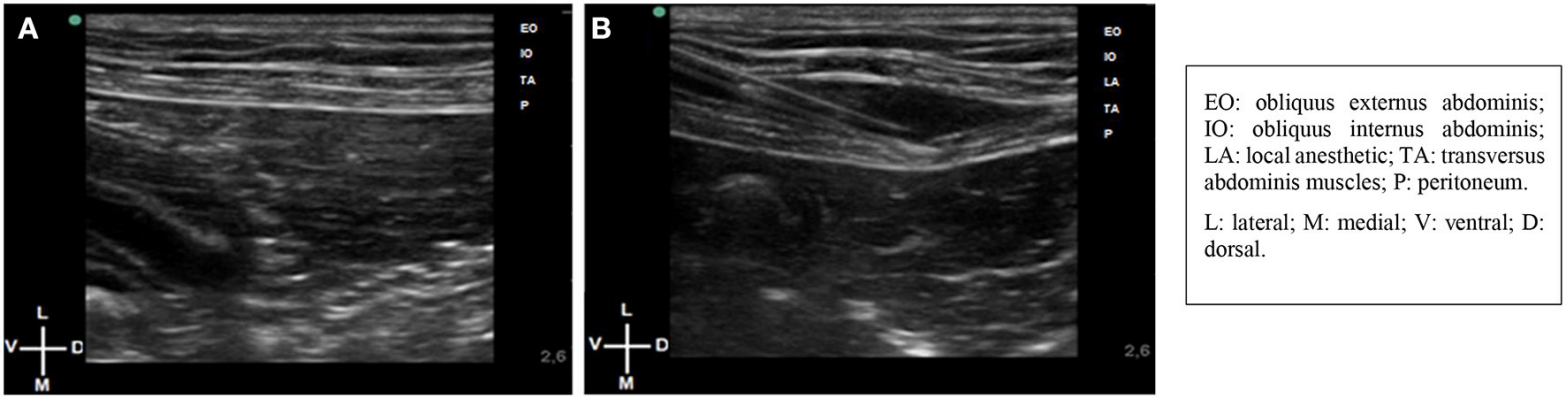
**(A)** Ultrasound images of the retrocostal region during the abdominal wall scanning in dog. **(B)** In plane technique. The Tuohy needle reaching the transverse abdominis plane, located between the obliquus internus abdominis and transversus abdominis muscles.

The hair of the entire abdominal wall was clipped and antiseptically prepared. Dogs were positioned in lateral recumbency with the side to be blocked uppermost. US gel (Aquasonic 100; Parker Laboratories Inc., NJ, USA) was applied to the transducer to facilitate acoustic coupling. An anesthetist experienced in US-guided regional anesthesia performed the procedure using a portable Sonosite M-Turbo (Sonosite Inc., WA, USA) and a linear array probe (13–6 MHz HLF-38; Sonosite Inc.). Injections were performed bilaterally at the retrocostal and preiliac regions (7). After identification of the three muscles layers (obliquus externus abdominis, obliquus internus abdominis, and transversus abdominis) and the peritoneum (Figure 1A), an 18–22-gauge Tuohy needle (Tuohy Epidural, Unilever Unisys, Japan) connected to the syringe was introduced “in-plane” in a ventrodorsally direction until reaching the interfascial plane, located between the obliquus internus abdominis and transversus abdominis muscles. Before injection, aspiration was performed, and after confirmation of a negative response, a test dose of 0.5 ml of water for injection was performed. After verification of the appropriate location and with the aid of a 3-way stopcock connected to the Tuohy needle, the injections were performed (Figure 1B). To access the contralateral hemiabdomen, recumbency was modified.

Surgery started 30 min after the TAP injection. To guarantee the same level of surgical stimulation, surgery was always performed by the same surgeon, with a midline incision using the same technique in all patients.

### Physiological variables

During the anesthetic procedure, patients were monitored with a multiparametric monitor (Nihon Kohden, Monitor Life Scope, MU-671 RK, Tokyo, Japan) continuously with the assessment of HR, peripheral oxyhemoglobin saturation (SpO_2_), respiratory rate (*f*_R_), esophageal temperature and invasive blood pressure (IBP) through the introduction of an appropriately sized catheter in the dorsal pedal artery. The pulse pressure variation (PPV) value was used to guide the management of intraoperative hypotension. Accordingly, if mean arterial pressure was below 60 mmHg and the PPV was more excellent than 16%, a fluid bolus of 15 ml kg^−1^ was started and administered in 15 min; however, if the PPV value was < 16%, hypotension was treated with ephedrine (0.1 mg kg^−1^) IV.

After the beginning of the surgical procedure, if HR and MAP increased by 20% concerning the values recorded at the start of remifentanil infusion, rescue analgesia was performed with a gradual increase (0.05–0.1 μg kg^−1^ min) in the infusion rate of remifentanil.

In the immediate post-operative period, the animals were sent to the post-anesthetic recovery room (PARR), where they were evaluated regarding pain by a no-blind observer. If necessary, the analgesic rescue was performed, which was morphine 0.1 mg kg^−1^ IV.

### Pain score and sedation degree

The same blind researcher evaluated the animals before applying pre-anesthetic medication and 1, 2, 4, 6, and 8 h after extubation of the patient. The NRS and the GCMPS-SF scale were used for pain assessment. Sedation was evaluated using the scale proposed by Wagner and collaborators (24). Rescue pain relief in the post-operative period consisted of administering 0.1 mg kg^−1^ of IV morphine to animals with an NRS ≥4 or GCMPS-SF ≥6. Animals were reassessed 30 min later, and if necessary, an additional 0.1 mg kg^−1^ IV was administered until adequate pain management.

Heart rate, *f*_R_, IBP, esophageal temperature, SpO2, end-tidal carbon dioxide, and isoflurane concentration, as mentioned before, were measured continuously during the surgical procedure and, for means of statistical analysis, the following time points were recorded: after 5 min of start of remifentanil infusion (RI), beginning of the surgical incision (SI), access and exposure of the right ovary (RO), access and exposure of the left ovary (LO), end of the surgical procedure (ES), which was considered at the beginning of the skin suture.

In the pre and post-operative period, the same variables were evaluated except for blood pressure that was non-invasively and the rectal temperature. Data were recorded at the following time points: before pre-anesthetic medication (Baseline) and then at 1, 2, 4, 6, and 8 h after extubation.

In addition, samples were taken to determine serum cortisol levels at the following time points: Baseline, RO, 2, 6, and 8 h after extubation. The analysis of serum cortisol was performed in duplicate through specific kits (ImmuChemTM Cortisol Coated Tube 125I RIA Kit, MP Biomedicals LLC, Solon, OH) by the radioimmunoassay method, and the results were recorded in μg/dl.

### Assessment of adverse effects

The adverse effects of the TAP injection and episodes such as nausea, emesis, and salivation were also recorded in the post-operative evaluation for later comparison.

### Post-operative analgesia

At the end of the 6-h evaluation period, patients received dipyrone (Febrax^®^, Lema Biologic do Brasil LTDA, Juatuba, MG) (25 mg kg^−1^) and meloxicam (Maxicam 2%, Orofino Saúde Animal Ltd., Cravinhos, SP) (0.1 mg kg^−1^), IV. The discharge occurred 8 h after extubation if the animals were pain-free and presented all clinical variables within the normal range.

### Statistical analysis

Calculating the sample size resulted in at least fourteen dogs based on the NRS and need for rescue medication of 85 and 25% for the control and bupivacaine group, respectively, with an 80% chance as significant at the 5% level.

Statistical analyzes were performed using GraphPad Prism version 6.0 (GraphPad Software Inc., La Jolla, EUA). Shapiro-Wilk test was used to assess the normality of the data, and to evaluate if the variances were equal, we employed the Barlett's test. For the analysis of weight, age, and surgical time, the unpaired *T*-test was used, and for physiological variables, two-way analysis of variance (ANOVA) with Sidak's post-test (between groups) and analysis of variance of repeated measures (RM—ANOVA) with Dunnett's post-test (between times).

Pain and sedation scores were compared between groups using the Kruskal-Wallis non-parametric test, followed by Dunn's post-test, and between the time points, the Friedman test was used, followed by Dunn's post-test.

The analgesic rescue was assessed between groups using Fisher's test, and animals that required analgesic rescue were not excluded from statistical analysis after that.

In addition, post-operative evaluations were compared with pre-operative baseline values. Finally, intraoperative variables were compared with the values recorded in RI, that is, after 5 min at the beginning of remifentanil infusion.

The level of significance considered was 5% (*p* < 0.05) for the tests performed.

## Results

Thirty-five female dogs were initially selected; however, three had to be excluded. One was excluded due to the change from ovariectomy to ovariosalpingohisterectomia, and the other two were for hostile behavior. In total, 32 healthy dogs equally distributed in groups TBC and TBB were included in the study.

There was no significant difference between groups regarding age (TBC group 17 ± 8.21 months and TBB group 21 ± 12.98 months), weight (TBC group 15 ± 7.47 kg and TBB group 14 ± 6.68 kg), and the duration of surgery (TBC group 44 ± 12.95 min and TBB group 47 ± 13.76 min).

Data in detail of the HR, *f*_R_, IBP, SpO_2_, P_E_'CO2, F_E_'Iso, and esophageal temperature during the surgical period and HR, *f*_R_, SAP, and rectal temperature during the post-operative period are presented in Tables 1, 2, respectively.

**Table 1:** Mean ± standard deviation of heart rate (HR), respiratory rate (*f*_R_), systolic arterial pressure (SAP), mean arterial pressure (MAP), diastolic arterial pressure (DAP), esophageal temperature (ET), peripheral oxygen saturation (SpO_2_), end-tidal carbon dioxide concentration (P_E_'CO_2_), and end-tidal isoflurane concentration (F_E_'Iso) in dogs during ovariohysterectomy.

**Parameter**	**Groups**	**RI**	**SI**	**RO**	**LO**	**ES**
HR (beats min^−1^)	TBC	100 ± 20.00	96 ± 27.55	97 ± 21.54 *f*	93 ± 22.50	93 ± 21.92 *f*
	TBB	92 ± 16.11	93 ± 19.09	75 ± 21.88	77 ± 16.25	74 ± 17.32*
*f*_R_ (breaths min^−1^)	TBC	11 ± 2.66	10 ± 2.13	10 ± 2.45	11 ± 2.45	11 ± 2.46
	TBB	11 ± 2.65	11 ± 2.53	11 ± 2.39	11 ± 2.82	11 ± 2.87
SAP (mmHg)	TBC	113 ± 23.31	108 ± 20.98	124 ± 13.82	114 ± 16.82	100 ± 16.25
	TBB	97 ± 16.14	108 ± 21.75	118 ± 29.11*	111 ± 23.54	100 ± 20.44
MAP (mmHg)	TBC	78 ± 16.92	72 ± 16.47	88 ± 13.06*	79 ± 13.76	69 ± 13.49
	TBB	67 ± 11.41	71 ± 13.59	86 ± 23.23*	78 ± 16.52*	66 ± 12.40
DAP (mmHg)	TBC	65 ± 15.94	60 ± 14.35	75 ± 13.25	67 ± 12.97	58 ± 12.70
	TBB	56 ± 10.86	62 ± 16.61	75 ± 22.46*	65 ± 15.65	55 ± 12.56
ET (°C)	TBC	35.2 ± 0.65	35 ± 0.62	34.8 ± 0.74	34.3 ± 0.83*	33.9 ± 0.84*
	TBB	35.5 ± 0.94	35.1 ± 1.06	34.8 ± 1.21	34.4 ± 1.23*	34 ± 1.13*
SpO_2_ (%)	TBC	100 ± 0.5	100 ± 0.5	100 ± 0.3	100 ± 0.5	100 ± 0
	TBB	100 ± 0.3	100 ± 0.3	100 ± 0.9	100 ± 0.5	100 ± 0.3
P_E_'CO_2_ (mmHg)	TBC	38 ± 4.23	39 ± 3.95	38 ± 4.35	38 ± 5.12	37 ± 3.75
	TBB	37 ± 6.53	36 ± 4.48	38 ± 4.65	40 ± 4.01	41 ± 2.66*
F_E_'Iso (%)	TBC	1.1 ± 0.19	1.1 ± 0.12	1.1 ± 0.08	1.1 ± 0.09	1.1 ± 0.11
	TBB	1.1 ± 0.14	1.1 ± 0.12	1.1 ± 0.09	1.1 ± 0.09	1.1 ± 0.08

*f* significantly different from the TBB group (p < 0.05); ^*^ significantly different from RI (p < 0.05).

RI, start of remifentanil infusion; SI, surgical incision; RO, exposure of the right ovary; LO, exposure of the left ovary; ES, end of the surgical.

Besides isoflurane, remifentanil constant rate infusion was used during the surgical procedure. Data are from 16 dogs in each group.

**Table 2:** Mean ± standard deviation referring to heart rate, respiratory rate, systolic blood pressure and rectal temperature in the post-operative period of the 32 animals in the two study groups during the evaluated times.

**Parameter**	**Groups**	**Baseline**	**1**	**2**	**4**	**6**	**8**
HR (bpm)	TBC	131 ± 27.66	116 ± 38.42	96 ± 21.54*f	98 ± 16.52*	99 ± 21.77*	101 ± 22.60*
	TBB	151 ± 28.62	126 ± 30.53*	122 ± 23.25*	121 ± 20.38*	108 ± 20.75*	110 ± 18.87*
*f*_R_ (mpm)	TBC	40 ± 16.62	20 ± 6.83*	20 ± 6.32*	22 ± 10.73*	22 ± 8.39*	22 ± 6.53*
	TBB	49 ± 12.78	23 ± 6.49*	24 ± 7.51*	25 ± 10.60*	24 ± 7.28*	22 ± 7.98*
SAP (mmHg)	TBC	151 ± 26.91*f*	160 ± 23.76	152 ± 26.08	157 ± 34.13	150 ± 22.92	147 ± 26.95
	TBB	177 ± 27.74	161 ± 29.49	160 ± 18.07	169 ± 31.35	163 ± 19.61	160 ± 22.12
T (°C)	TBC	38.7 ± 0.54	37.2 ± 0.74*	37.7 ± 0.57*	38.1 ± 0.44*	38.1 ± 0.41*	38.2 ± 0.34*
	TBB	38.8 ± 0.32	37.2 ± 0.53*	37.7 ± 0.52*	38.1 ± 0.48*	38.3 ± 0.38*	38.2 ± 0.28*

*f* significantly different from the TBB group (p < 0.05);

^*^ significantly different from baseline (p < 0.05); 1, 2, 4, 6, and 8 h after extubation.

### Assessment of intraoperative pain

Data in detail of the HR, *f*_R_, IBP, SpO_2_, P_E_'CO2, F_E_'Iso, and esophageal temperature are presented in Table 1.

The doses of remifentanil infusion were increased from 0.2 to 0.3 μg kg^−1^ min in three animals from the TBC group and two animals from the TBB groups. Still, there is no significant difference in the number of dogs in the two groups requiring this adjustment.

In the intraoperative period, four animals in the TBC group had hypotension which was treated with a fluid challenge (15 ml kg^−1^ in 15 min). One animal, besides hypotension, had bradycardia managed with the stop of remifentanil infusion and administration of atropine.

**Figure 2:**
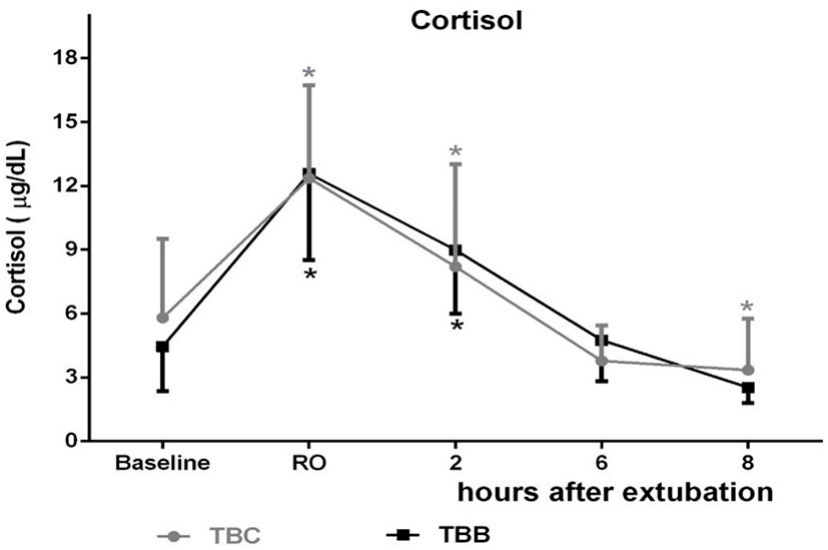
Mean ± standard deviations of serum cortisol, in micrograms per deciliter (μg/dl), of the 32 animals in the two study groups during the evaluated times. * significant difference from baseline.

Although the serum cortisol levels showed no significant difference between groups, there is a substantial increase in moment RO when compared to baseline in the TBC group (12.36 ± 4.36 μg/dl*; p* < 0.0001) and in the exact moment in the TBB group (12.58 ± 4.07 μg/dl; *p* < 0.0001) (Figure 2).

### Assessment of post-operative pain

Data in detail of the HR, *f*_R_, SAP, and rectal temperature during the post-operative period are presented in Table 2.

**Figure 3:**
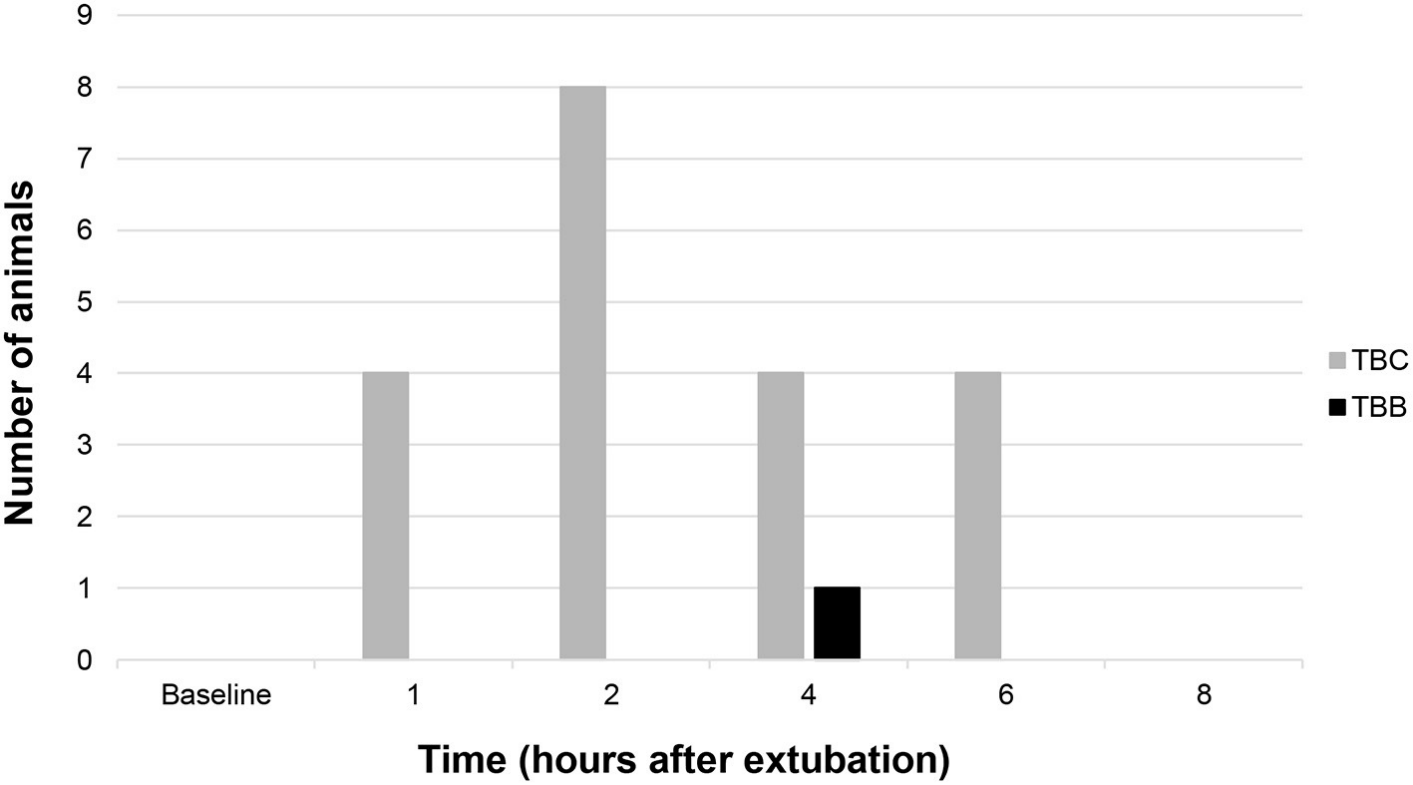
Number of animals rescued with morphine in both groups in the post-operative period.

There was a significant difference between the groups (*p* < 0.0001) regarding the need for rescue medication in the post-operative period, with the TBC group needing rescue medication in 13 animals and the TBB group in only one animal, according to NRS and GCMPS-SF scale (Table 3, Figure 3).

**Table 3:** Pain score and sedation score presented as median (minimum-maximum) of the 32 animals randomly distributed in the two study groups during the evaluated times.

	**Groups**	**Baseline**	**1**	**2**	**4**	**6**	**8**
NRS	TBC	0 (0–0)	3 (1–4)*	3 (1–4)*	2(1–4)*	2 (0–3)*	0.5 (0–1)
	TBB	0 (0–0)	2 (1-3)*	2 (1–3)*	1 (0–4)*	1 (0–2)*	0 (0–1)
GCMPS-SF	TBC	1 (0–2)	4 (1–14)*	5 (1–8)*	3.5 (0–11)*	3.5 (1–8)*	1 (0–5)
	TBB	1 (0–2)	3 (0–5)	3 (0–5)	1 (0–6)	1 (0–5)	0 (0–1)
Sedation score	TBC	0 (0–1)	4.5 (3–6)*	4.5 (3–7)*	3 (2–9)*	3.5 (3–5)*	3 (0–3)
	TBB	0 (0–2)	5 (3–8)*	4 (3–7) *	3 (3–5)*	3 (1–3)*	3 (1–3)

GCMPS-SF, Glasgow composite measure pain scale; NRS, numeric rating scale.

^*^ significantly different from baseline (p < 0.05) 1, 2, 4, 6, and 8 h after extubation.

Regarding the sedation score, there was no difference between groups (Table 3).

The adverse effects were observed in five animals in the TBC group and four animals in the TBB group who presented emesis in the first 2 h post-operatively.

Cortisol increased significantly 2 h after extubation compared to baseline in the TBC group (8.20 ± 4.82 μg/dl; *p* = 0.0441) and the TBB group (8.99 ± 3.00 μg/dl; *p* < 0.0001) when compared to the baseline. Furthermore, in the TBC group, there is a decrease 8 h after extubation compared to baseline (3.34 ± 2.42μg/dl; *p* = 0.0384).

## Discussion

This study showed that administering 0.2 ml kg^−1^ of 0.25% bupivacaine in the four TAP block points in dogs submitted to ovariectomy promoted adequate post-operative analgesia, significantly reducing the consumption of systemic analgesics for at least 6 h after extubation. This result corroborates with the studies showing the tap block's analgesic efficacy in the immediate post-operative period in dogs for mastectomy (3, 16), cats submitted to ovariectomy (5) and in dogs undergoing elective ovariohysterectomy (17).

There are different approaches to performing the TAP block, and the choice will depend on the type of surgery. The retrocostal and preiliac methods utilized in the present study are indicated when the surgical procedure involves the midline according to those described by Portela et al. (3) in dogs and Skouropoulou et al. (5) in cats.

Freitag (25) showed that the TAP block, associated with the superficial serratus plane block in female dogs submitted to mastectomy, could be performed between the two points. The ultrasound transducer is positioned parallel to the ribs at the midpoint between the last rib and the iliac crest anterior margin. With this approach, animals showed adequate post-operative analgesia for up to 24 h. Another study also showed that inserting a catheter at the mid-abdominal point could be effective for analgesia in two dogs with pancreatitis and one for an exploratory laparotomy performed to remove a mass enveloping the spleen and pancreas (4).

Theoretically, the two-point approach could make the procedure faster and effortless, but the spread is different. In a literature review (26), the authors suggested that the subcostal point's approach allows the local anesthetic to reach the thoracic vertebrae in T10, T11, T12, and T13, and the mid-abdominal end goes from T13, L1, and L2. In an anatomical evaluation with Beagle cadavers, 10 ml of a mixture of methylene blue and bupivacaine midway between the iliac crest and the caudal aspect of the rib cage effectively stained the following nerves T11 (20%), T12 (60%), T13 (100%), L1 (100%), L2 (90%), and L3 (30%) (10). However, to our knowledge, there are no studies for ovariectomy in dogs using the two-points approach by hemiabdomen or four-points approach technique. Considering that the surgical procedure involves the midline, we chose this approach.

Regarding the local anesthetic, duration, dilution, and volume to be used, there is still no consensus in the literature on the TAP block. However, according to Otero and Portela (27), bupivacaine used at the concentration of 0.5% can reach up to 5–8 h for mixed blocks and 6–18 h for a sensory block. On the other hand, when lower concentrations are used, both the intensity and duration of the blockade decrease and the chance of toxicity. For this reason, we chose to utilize 0.25% to avoid possible toxicity of the local anesthetic, as the total volume to perform the block bilaterally would be relatively high. Thus, we worked with 2 mg kg^−1^ as it is the maximum dose recommended for bupivacaine (27) with a volume of 0.2 ml kg^−1^, 0.25% in each point. In cats submitted to ovariectomy, the blockade was performed with a mixture of bupivacaine at 0.5% at 0.2 ml kg^−1^ diluted to a total volume of 1.5 ml in 2% lidocaine bilaterally, promoting adequate analgesia for up to 24 h post-operatively (5). However, in the former study, the administration of robenacoxibe and methadone probably contributed to the enhanced analgesia length observed (24 h).

In the present study, only pethidine and remifentanil were used during the perioperative period to not interfere with the post-operative evaluation, considering that remifentanil is a short-acting opioid. Likewise, the high number of animals needing rescue analgesia in the control group confirms that both pethidine and remifentanil probably did not interfere with the degree of post-operative analgesia verified in the bupivacaine group.

During the intraoperative period, the control group showed higher heart rate values than the bupivacaine group during the RO time point. We could argue that this result could signal that the block effectively provided some visceral analgesia for these dogs because both groups received the same remifentanil CRI. Also, the number of dogs receiving an increase in this CRI was the same for the two groups. Nevertheless, blood pressure values and cortisol increased significantly when traction of the first ovary was performed in the two groups, which refutes this hypothesis. However, it was not the scope of this study to evaluate remifentanil consumption. On the contrary, in 66 women, Karam et al. (28) concluded that using TAP block for hysterectomy helped decrease the consumption of general anesthetics and opioids.

According to some authors, cortisol values can increase during surgical manipulation of the ovaries in female dogs (29–31) and during the post-operative period (32). In our study, as mentioned before, using remifentanil and the TAP block together did not abolish this response. Systemic opioids are usually ineffective in blunting the neuroendocrine response depending on the level of the surgical stimulation in dogs, as shown in orthopedic procedures and OSH (22, 33). In the human being, Møiniche et al. (34), in a systematic review, showed that only the use of epidural analgesia with opioids, local anesthetics, or a mixture promotes preemptive analgesia. On the other hand, peripheral blocks and spinal or epidural opioids can block this response (21, 22).

One study in dogs showed that the plasma cortisol concentrations only returned to baseline values 24 h after surgery (35). In another study, values did not return to baseline showing only decreased tendency (36). Although cortisol values did not return to baseline values in our study, there was a 42% and 43% decrease for the TBC and TBB groups, respectively, 8 h after extubation.

The present study was based on validated pain scales to perform the analgesic rescue medication since the results related to physiological variables assume that they are not good indicators for recognizing pain in dogs. After all, these variables can be influenced by factors other than pain, such as stress, and mainly due to the animal's behavior (37).

Thus, considering the scales used for pain assessment in the present study, both did not differ when assessed between groups. However, when compared with baseline time, the scores obtained by the NRS showed a difference in 1, 2, 4, and 6 h after extubation. This result can be justified because all patients were healthy and young, scoring zero at baseline. For the TBB group, the increase in scores never reached a value likely to receive a rescue medication except for one dog, which received rescue analgesia 4 h after extubation. However, 8 h after extubation, there was no difference compared to the baseline. Besides the rescues performed, when necessary, dipyrone and meloxicam were administered in all patients, which certainly may have contributed to this result.

The pain scores obtained by the GCMPS-SF scale showed a significant difference in the control group at 1, 2, 4, and 6 h after extubation compared with baseline. Thirteen animals were in the control group, while only one animal in the TBB group needed rescue analgesia. This result corroborates with studies that show the analgesic efficacy of the TAP block in the immediate post-operative period in small animals (3, 5, 15–17). However, the percentage of rescue medication in the study of ovariohysterectomy was higher than the percentage observed in the present study. In the group that received the association of bupivacaine and dexmedetomidine, 4 out of 9 animals needed rescue medication, whereas in the group treated with the liposomal bupivacaine, 3 out of 9 (17). In our study, rescue medication was required in 1 out of 16 animals. The methodology, dilutions, and drugs can explain the differences and associations employed.

Furthermore, there was a significant difference between 1, 2, 4, and 6 h after extubation in the post-operative sedation scores compared to baseline; this can be understood due to the residual effect of the drugs used in the protocol.

Because we were not sure about the exact duration of the block, all patients received dipyrone and meloxicam 6 h after extubation and were evaluated with pain and sedation scales before hospital discharge. Only one animal in the TBB group showed high pain scores needing rescue analgesia, demonstrating that our expectations regarding the effectiveness of the block were correct.

In fact, in the present study, all animals receiving bupivacaine in the TAP block showed an effective block, demonstrated by the degree of analgesia obtained in the post-operative period and the low rate of rescue analgesia needed (1 out of 16 in the TBB, against 13 out of 16 in the TBC).

As the limitation of this study, we can mention the small number of animals compared to human studies. Also, an experienced surgeon performing the surgical procedure could minimize the pain in both groups. Although we kept the animals on the statistical analysis after the analgesic rescue, which undoubtedly influenced pain scores, the result was supported by evaluating the number of animals needing further analgesia. Since the study's main objective was to evaluate the analgesic efficacy of the TAP block, the sample size was calculated based on rescue analgesia. Probably this choice would not be the best option regarding the secondary objectives, mainly the cortisol analysis.

## Conclusion

In conclusion, the technique using ultrasound-guided TAP block in two points approach by hemiabdomen with 0.2 ml kg^−1^ bupivacaine 0.25% was effective in providing post-operative analgesia in dogs undergoing ovariectomy. In both groups, the MAP value increased during surgical stimulation in a very similar way. This finding supports the hypothesis that the TAP block is not effective in blocking visceral nociceptive stimulation. In contrast, the clinical utility of the TAP block is evident for the post-operative period. This finding should be underlined in the conclusion of the study. Although the neuroendocrine response was not modulated during the intraoperative period, the cortisol went down 6 and 8 h in the post-operative period. Furthermore, the TAP block was capable of diminished analgesic rescue, as hypothesized.

## Data availability statement

The original contributions presented in the study are included in the article/supplementary material, further inquiries can be directed to the corresponding author/s.

## Ethics statement

The animal study was reviewed and approved by Ethic Committee on Animal Use of the School of Veterinary Medicine and Animal Science, University of São Paulo, Brazil. Written informed consent was obtained from the owners for the participation of their animals in this study.

## Author contributions

JC: study design, data management, interpretation, and manuscript preparation. PO: study design, data management and interpretation, preparation of the manuscript, and English review. AA: data interpretation, statistical analysis, manuscript preparation, and English review. IN and FP: data management. MP: data interpretation and statistical analysis. JM: performed all the surgeries and study design. DF: study design, funding obtention, data management and interpretation, preparation of the manuscript, and English review. All authors read and approved the final manuscript.

## Funding

This work was carried out with the support of the Coordination of Improvement of Higher Education Personnel, Brazil (CAPES), Financing Code 001.

## Conflict of interest

The authors declare that the research was conducted in the absence of any commercial or financial relationships that could be construed as a potential conflict of interest.

## Publisher's note

All claims expressed in this article are solely those of the authors and do not necessarily represent those of their affiliated organizations, or those of the publisher, the editors and the reviewers. Any product that may be evaluated in this article, or claim that may be made by its manufacturer, is not guaranteed or endorsed by the publisher.
